# An oncogenic role for microRNA-146b in the thyroid

**DOI:** 10.18632/oncoscience.432

**Published:** 2018-06-27

**Authors:** Julia Ramírez-Moya, Pilar Santisteban

**Affiliations:** Instituto de Investigaciones Biomédicas, CSIC-UAM, Ciberonc, Madrid, Spain

**Keywords:** microRNA146b, PTEN, thyroid cáncer

There is a wealth of evidence supporting the deregulation of microRNA (miR) expression in the hallmarks of cancer, including sustaining proliferative signaling, resisting cell death, or activating invasion and metastasis. In the last years, many studies have been published identifying miRs as potential biomarkers for cancer diagnosis and prognosis as well as therapeutic targets, adding an extra dimension to cancer evaluation and treatment. Nevertheless, further investigation is required to understand how miRs influence tumorigenesis by acting as tumor suppressors and oncogenes. Thyroid cancer constitutes an excellent model for the exploration of these mechanisms as the biology underlying its initiation and progression has been studied extensively [1].

Thyroid tumors constitute the most frequent endocrine tumors and are classified into different types based on histological architecture and cellular morphology. Papillary and follicular thyroid carcinomas (PTC and FTC), which are well-differentiated tumors, account for the majority of thyroid cancer, whereas poorly differentiated and anaplastic thyroid carcinomas (PDTC and ATC) are the rarest and most aggressive forms. Although thyroid cancer is the fastest growing cancer worldwide, most are treatable by surgery, hormone therapy and radioactive iodine.

Initiation and progression of thyroid cancer is marked by multiple genetic alterations, of which mutations leading to the activation of MAPK and PI3K-AKT signaling are the most studied. PI3K/AKT activation is critical in FTC initiation through mutations in RAS, inactivating mutations in the tumor suppressor *PTEN*, or activating mutations in *PIK3CA* and *AKT1*. Some patients with PTC and FTC will ultimately progress to metastatic disease, and these tumors are particularly enriched with mutations in *BRAF* and *RAS* coexisting with *PIK3CA* or *AKT1* mutations [2, 3].

The Cancer Genome Atlas (TCGA) [4] has identified thousands of mutations in human cancer, and has provided new insights into the role of mutated genes and the genetic alterations that lead to disease development. Advances in the study of PTC [4, 5], and ATC [6], have paved the way to a better understanding of the genomic and transcriptomic landscape of thyroid cancer. Accordingly, the role of miRs in the control of cancer gene expression is evolving as an important mechanism as they function to destabilize mRNAs and to inhibit translation through binding to the 3′-untranslated region (3′-UTR) of target mRNAs. The role of specific miRs in thyroid carcinogenesis is well established both clinically and experimentally. Among those abundantly overexpressed in thyroid cancer, miR-146b (5p and 3p) is considered a prognostic factor for PTC.

We previously demonstrated that miR-146b-3p specifically represses, in a feedforward loop, the transcription factor PAX8 and its target gene the sodium iodide symporter (NIS), whose activity forms the basis for radioiodine therapy [5]. Accordingly, overexpression of miR-146b in thyroid cancer represses the thyroid differentiation phenotype and could desensitizes patients to conventional radioiodine-based therapy. In our new study [7], we found that PTEN contains a functional miR-146b-5p binding site in its 3′-UTR. Overexpression of miR-146b in thyroid cells significantly decreased PTEN expression and increased AKT activation, demonstrating that miR-146b activates PI3K signaling by silencing PTEN. By analyzing the components downstream of PI3K/AKT, we observed that miR-146b induced FOXO1 and p27 nuclear exclusion with the consequent increase in thyroid cell proliferation. Moreover, miR-146b overexpression protected cells from apoptosis by elevating the levels of Bcl-2 and pBAD proteins. Finally, miR-146b increased migration and invasion of thyroid cells and regulated important genes involved in epithelial-mesenchymal transition, such as Twist and E-cadherin.

A central finding of our study was that the effects triggered by miR-146b activation were rescued, at least in part, by exogenous expression of PTEN. Accordingly, PTEN overexpression rescued the miR-146b-induced effects on cell cycle progression, invasion and migration and survival. Interestingly, PTEN overexpression failed to rescue E-cadherin levels, suggesting that the effect of miR-146b on this epithelial cell-adhesion molecule is not mediated via PI3K. Indeed, we found that miR-146b inhibited E-cadherin expression through binding to its 3′UTR, representing another new target for this oncogenic miR.

An important translational aspect of our study was the finding that miR-146b inhibition using a synthetic and clinically amenable molecule decreased miR-146b-induced cancer aggressiveness. Thus, intratumoral delivery of a miR-146b antagomiR blocked tumor growth *in vivo* in a human thyroid xenograft model. As anticipated, this inhibition resulted in the activation of the two target genes PTEN and E-cadherin. Overall, these data provide support for the use of mechanism-based miR inhibitors as therapeutic options to target advanced thyroid cancer.

**Figure 1:**
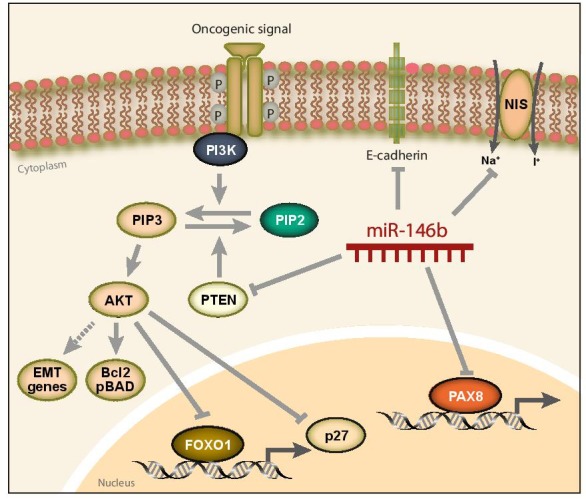
Model explaining the diverse oncogenic actions of miR-146b in thyroid cancer

Our work demonstrates that the overexpression of miR-146b in thyroid cancer is a bona fide oncogenic event since it targets not only genes involved in differentiation, such as PAX8 and NIS, but it also abolishes the expression of the tumor suppressor PTEN and the epithelial marker E-cadherin. PTEN inhibition leads to PIP3 accumulation, thus hyperactivating the PI3K signaling pathway that, in turn, represses p27 and FOXO1 function while enhancing BCL-2, pBAD, and EMT gene expression. As a consequence of these events, tumors with elevated miR-146b levels will be resistant to radioiodine treatment, and will have constitutive activation of the PI3K pathway, resulting in a more mesenchymal phenotype. Therefore, these tumors will have a worse prognosis and will be likely to progress from papillary to advanced thyroid tumors (Figure 1).
